# ASO Author Reflections: Does the Axilla Still Matter? Rethinking Nodal Staging in the Era of CDK4/6 Inhibition

**DOI:** 10.1245/s10434-026-20171-9

**Published:** 2026-07-06

**Authors:** Austin D. Williams

**Affiliations:** Division of Breast Surgery, Department of Surgery, Fox Chase Cancer Center, Philadelphia, PA USA

## Past

Axillary staging has long been central to the management of early-stage breast cancer, providing prognostic information and guiding adjuvant therapy decisions. Over time, the extent of surgery has progressively decreased from routine axillary lymph node dissection to sentinel lymph node biopsy (SLNB), whereas nodal status has remained a key determinant of treatment escalation. However, the growing use of genomic assays has already begun to shift this paradigm, particularly for postmenopausal patients, with recurrence scores currently guiding adjuvant chemotherapy decisions even in the setting of limited nodal involvement. More recently, the introduction of adjuvant cyclin-dependent kinase 4/6 (CDK4/6) inhibitors has expanded therapeutic options for patients with higher-risk hormone receptor-positive (HR+)/human epidermal growth factor receptor 2-negative (HER2–) disease, with eligibility often defined by nodal involvement.^1^^,^^2^ As evidence supporting omission of SLNB has emerged, an important clinical question has followed: does forgoing nodal staging compromise the ability to identify patients who may benefit from these newer systemic therapies?

## Present

Prior work has demonstrated that axillary lymph node dissection should not be performed solely to determine candidacy for adjuvant abemaciclib given its low yield and substantial morbidity.^3^^,^^4^ At the same time, the recent approval of ribociclib has broadened eligibility for CDK4/6 inhibition beyond strictly node-positive disease, further diminishing reliance on nodal status for treatment selection. Together, these developments call into question the continued role of axillary staging in guiding systemic therapy and shift attention to a more immediate issue: whether SLNB remains necessary in this evolving therapeutic landscape.

In this context, the findings of the current study suggest that the incremental value of SLNB for determining CDK4/6 inhibitor eligibility is limited.^5^ Although approximately 10% of patients harbor nodal disease, omission of SLNB would exclude only a small minority of patients from meeting CDK4/6 eligibility criteria. Moreover, the clinical impact of the knowledge of nodal positivity appears modest, with roughly 300 SLNBs required to identify a single patient expected to derive a 5-year invasive disease-free survival benefit. These observations are consistent with prior single-institution studies evaluating the same question, which similarly demonstrated that relatively few omission-eligible patients are newly identified as candidates for CDK4/6 inhibition based on nodal findings. Taken together, these data suggest that the contribution of SLNB to systemic therapy decision-making is diminishing, particularly as treatment paradigms increasingly prioritize tumor biology and genomic risk over anatomic staging.

## Future

In this era of de-escalation, the goal is to minimize morbidity while maintaining or improving oncologic outcomes. As systemic therapies evolve, reliance on nodal status alone to guide treatment selection will continue to diminish, with increasing emphasis on tumor biology and genomic risk. At the same time, emerging data add important nuance. Adjuvant abemaciclib has demonstrated a modest overall survival benefit in high-risk populations, whereas long-term survival data for ribociclib remain immature. As these data mature, future studies must better define the magnitude of survival benefit and weigh it against the morbidity of surgical staging.

Multidisciplinary collaboration will be central to right-sizing breast cancer treatment. Trials of de-escalation must account for parallel advances across surgical, medical, and radiation oncology instead of evaluating each in isolation. Without this alignment, there is a risk of both unintended consequences and gaps in knowledge that make patient care more challenging. These trade-offs will increasingly require patient-centered decision-making, as individuals define their own thresholds for morbidity and oncologic benefit. Without coordinated trial design, however, the data needed to support these decisions will remain limited.
